# Subarachnoid Space: New Tricks by an Old Dog

**DOI:** 10.1371/journal.pone.0037529

**Published:** 2012-05-31

**Authors:** Andrzej F. Frydrychowski, Arkadiusz Szarmach, Bartosz Czaplewski, Pawel J. Winklewski

**Affiliations:** 1 Institute of Human Physiology, Medical University of Gdansk, Gdansk, Poland; 2 Department of Radiology, Medical University of Gdansk, Gdansk, Poland; 3 Department of Teleinformation Networks, Gdansk University of Technology, Gdansk, Poland; University of Maryland, United States of America

## Abstract

**Purpose:**

The purpose of the study was to: (1) evaluate the subarachnoid space (SAS) width and pial artery pulsation in both hemispheres, and (2) directly compare magnetic resonance imaging (MRI) to near-infrared transillumination/backscattering sounding (NIR-T/BSS) measurements of SAS width changes in healthy volunteers.

**Methods:**

The study was performed on three separate groups of volunteers, consisting in total of 62 subjects (33 women and 29 men) aged from 16 to 39 years. SAS width was assessed by MRI and NIR-T/BSS, and pial artery pulsation by NIR-T/BSS.

**Results:**

In NIR-T/BSS, the right frontal SAS was 9.1% wider than the left (p<0.01). The SAS was wider in men (p<0.01), while the pial artery pulsation was higher in women (p<0.01). Correlation and regression analysis of SAS width changes between the back- and abdominal-lying positions measured with MRI and NIRT-B/SS demonstrated high interdependence between both methods (r = 0.81, p<0.001).

**Conclusions:**

NIR-T/BSS and MRI were comparable and gave equivalent modalities for the SAS width change measurements. The SAS width and pial artery pulsation results obtained with NIR-T/BSS are consistent with the MRI data in the literature related to sexual dimorphism and morphological asymmetries between the hemispheres. NIR-T/BSS is a potentially cheap and easy-to-use method for early screening in patients with brain tumours, increased intracranial pressures and other abnormalities. Further studies in patients with intracranial pathologies are warranted.

## Introduction

The subarachnoid space (SAS) refers to the space between the arachnoid and the pia mater, the innermost membrane surrounding the central nervous system. The arachnoid is named for its delicate, spider-web-like filaments that extend from its undersurface through the cerebrospinal fluid (CSF) in the subarachnoid space to the pia mater. “Arachnoid” comes from the Greek “arachne” meaning spider or cobweb and “eidos” meaning resemblance, which in turn translates into spider-web-like. The SAS has been measured by various methods in children, mostly by ultrasonography [1], [2]. Generally for infants, an SAS width less than 4 mm is considered normal [3]. Ventricular or SAS dilatation in children may be a marker for the development of several neuropsychiatric disorders, autism, mental retardation or schizophrenia [4], [5], to name a few. The SAS has been studied much less frequently in adults. Nevertheless, there is one study in alcohol-dependent patients, where the mean SAS width was very close to 3 mm in the control group consisting of 10 healthy subjects [6].

In the last decade, a new method based on infrared radiation (IR) called near-infrared transillumination/backscattering sounding (NIR-T/BSS) has been developed. NIR-T/BSS allows for measurement of the SAS width to determine changes in CSF volume and/or intracranial pressure [7]–[14]. Contrary to near-infrared spectroscopy (NIRS), which relies on the absorption of IR by haemoglobin [15], [16], NIR-T/BSS uses the SAS filled with translucent CSF as a propagation duct for IR. In addition, NIR-T/BSS enables the assessment of changes in pial artery pulsation [10], [12]–[14], [17], [18]. Due to its non-invasive characteristic, ease of use and low cost, NIR-T/BSS constitutes a potential tool for screening changes in the SAS width and pial artery compliance caused by intracranial pathologies such as brain tumours and early brain oedema.

It can be assumed that the elevated interstitial fluid pressure of human tumours [19] and compression on the surrounding brain should affect the SAS, and cause asymmetries between the left and right hemispheres. Furthermore, studies using dynamic contrast enhanced magnetic resonance imaging (MRI) suggest that particular types of brain tumours, like high-grade gliomas, are associated with increased brain vasculature permeability and flow [20]. Such changes affecting brain microcirculation compliance can also potentially be detected by NIR-T/BSS. The purpose of the current study was to: (1) evaluate SAS width and pial artery pulsation in both hemispheres, including potential asymmetries, and (2) directly compare MRI and NIR-T/BSS measurements of SAS width in healthy volunteers. Therefore, in broader terms, the study was designed to establish a reference for future MRI versus NIR-T/BSS head-to-head comparison in patients with brain malignancies.

## Materials and Methods

The experimental protocol and the study were approved by the ethical committee of the Medical University of Gdansk (TKEBN 383/2008). The study was performed on three separate groups of volunteers. No coffee, food or nicotine was permitted for 3 hours before the test. Additionally, prior to the test, the volunteers were asked to sit comfortably and rest for 30 minutes. Subjects from the second and third groups did not have any disorders and were not taking any medications. The volunteers were selected on the basis of a medical questionnaire, interview and neurological examination. All of them gave written informed consent to participate in the study. In case of adolescent patients in addition the written informed consent from carers or guardians was obtained. The first group consisted of fifteen patients (9 women and 6 men) aged 16 to 39 years who were advised to undergo MRI examination because they suffered from headaches and tinnitus. However, according to conventional MRI examinations, the patients showed no brain pathology and were considered healthy. In this group of patients, only an MRI examination was performed. The second group consisted of 38 healthy volunteers (23 women and 15 men) aged 21 to 22 years. In this group of volunteers, only an NIR-T/BSS examination was performed in the rest sitting position and during the bend-over-position test (BOPT), when the subject bent forward at a 45°-angle between the trunk and the head [10], [14]. The third group consisted of 9 healthy volunteers (1 woman and 8 men), aged 24–31 years, who agreed to participate in simultaneous MRI and NIRT-B/SS measurements. In this group, MRI and NIR-T/BSS assessments were performed in the back- and abdominal-lying positions and the difference between the two positions was calculated. MRI measurements were performed by an experienced radiologist (AS), while NIR-T/BSS recordings were acquired by a different operator (AFF). To avoid any bias, AS and AFF did not see each other's results. All calculations and statistical analysis were performed by BC and PJW, who did not participate in data collection from the subjects.

Assessments of the subarachnoid space (SAS) were performed on an MRI system operating at 1.5 T (Picker Eclipse, NJ, US) with a standard, circularly polarised head coil. Fast spin-echo T2-weighted sequences were performed in the axial plane (6200/90 [TR/TE] images, a data matrix of 512×256 pixels, 24-cm field of view and 34 continual sections of 3-mm thickness, without intravenous contrast agent injection). SAS assessment was carried out in a single layer of the brain, above the lateral ventricles. Measurements were performed at the level of the superior frontal gyrus and occipital lobes at identical levels. The effects were achieved: (1) in the first group after four dimensions for each of the hemispheres of the brain (two frontal and two occipital lobes) and (2) in the third group after two dimensions for each of the hemispheres of the brain (two frontal lobes). Average values from three measurements of each dimension were taken for further analysis.

Bilateral changes in the width of the SAS and pial artery compliance were also recorded using a head-mounted NIR-T/BSS sensor unit of our own design. The sensor unit consisted of the emitter (E) and two photo-sensors located at various distances from the emitter. The NIR-T/BSS emitter was a light-emitting diode (LED). The proximal sensor (PS) was located close to the emitter, while the distal sensor (DS) was located further from the emitter. The stream of IR generated by the emitter penetrates the highly perfuse layer of the skin of the head, the skull bones and the SAS. The stream of radiation reflects from the surface of the brain and reaches the sensors, crossing the aforementioned layers of tissues in reverse order. Signals from the sensors undergo analogue-digital conversion in a specialised data acquisition system, and are recorded on a microcomputer's hard disk for subsequent analysis with on-line computer presentation.

Theoretical and practical foundations of the NIR-T/BSS method were provided in the earlier model studies [7], [8], [10], [11]. Briefly, the signal received by the DS is divided over the signal received by the PS. Such a division reduces the proportional factors that affect each of the two signals in an identical way, due to the fact that the quotient of these factors assumes the value 1. Both the dividend, i.e., the power of the DS signal, and the divisor, i.e., the power of the PS signal, are influenced by the width of the SAS as well as by any factor capable of changing that width. Therefore, the quotient of the two signals, hereafter called the transillumination quotient (TQ), is sensitive to changes in the width of the SAS. The oscillations of TQ have their origin in different modulation of the PS and DS signals, namely in the modulation of the DS signal on its way through the SAS. This happens because only the DS receives the radiation propagated within the SAS. Propagation of IR in the skin and bone is much worse than in the clear, translucent CSF of the SAS, and with the DS placed far enough from the emitter, no radiation propagated in the superficial tissue layers can reach the DS [7], [8]. The power of the IR stream reaching the DS is directly proportional to the width of the SAS. The wider the SAS or the propagation duct, the more radiation reaches the DS and the greater the signal from that sensor, which is the dividend in the calculation of the TQ [7], [8].

Thus, in the transillumination quotient (TQ), three main components can be identified:

1. a constant or non-pulsatile component, further referred to as sas-TQ, its value depending on the permeability of radiation through the skin and bones, as well as on the width of the CSF-filled SAS,
2. slow-variable pulsation, further referred to as the subcardiac component (scc-TQ), mainly of respiratory origin,
3. fast-variable pulsation, further referred to as the cardiac component (cc-TQ), resulting from heart-generated arterial pulsation that causes the fast oscillations in the SAS width.

The first harmonic of the arterial pulsation-dependent oscillations of TQ is extracted through appropriate filtering, along with its modulation, for further analysis. Modulation of that harmonic is a fast-variable component (or cardiac component) of the principal, second and third harmonics of the cardiac component waveform, respectively. A detailed description of the method of signal analysis is presented in other papers [7], [8], [10], [11].

The statistical methodology section was combined with the results section to facilitate understanding.

## Results

### MRI

**Figure 1:**
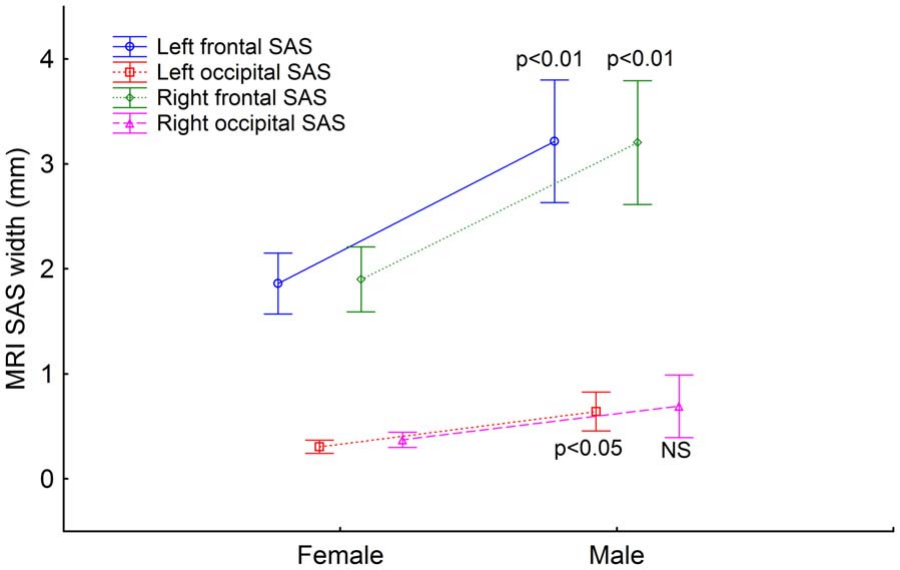
Dots and whisker plots show the mean left frontal (blue), right frontal (green), left occipital (red) and right occipital (purple) SAS width measured with MRI in females (n = 9) and males (n = 6). The dots represent the means and the whiskers the 95% confidence intervals. Statistical significance (p values or NS for not statistically significant) are for sex asymmetries (females vs. males).

The numerical values of the left and right SAS are presented in Table 1. Student's t-test and ANOVA were used for analysis of differences between average values. Weighted averages were used for sex difference comparisons. The right frontal SAS was 1.4% wider than the left, while the right occipital SAS was 8.8% wider than the left, although the differences remained statistically non-significant. Correlation and regression analysis revealed strong interdependences between the right and left SAS width: frontal r = 0.84; occipital r = 0.87; p<0.0001. Males had a significantly increased SAS width (Figure 1).

**Table 1:** Mean values and standard deviations of the SAS width measured with MRI (mm).

Subarachnoid space (MRI)	Number	Mean (mm)	Standard deviation (mm)
Right frontal	15	2.40	1.17
Left frontal	15	2.37^NS^	1.10
Right occipital	15	0.50	0.35
Left occipital	15	0.44^NS^	0.26

NS not statistically-significant difference versus right SAS.

### NIR-T/BSS (sas-TQ)

**Figure 2:**
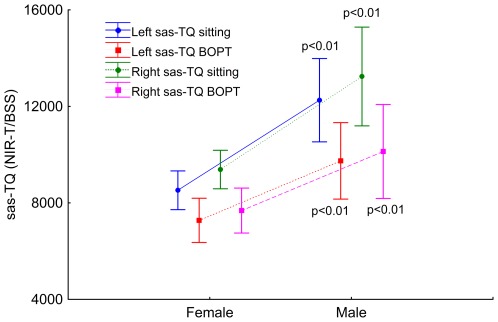
Dots and whisker plots show the mean left (blue) and right (green) frontal SAS width (sas-TQ) in the sitting position, and left (red) and right (purple) frontal SAS width (sas-TQ) during the BOPT measured with NIR-T/BSS in females (n = 23) and males (n = 15). The dots represent the means and the whiskers the 95% confidence intervals. Statistical significance (p values or NS for not statistically significant) are for sex asymmetries (females vs. males).

The numerical values of the left and right SAS are presented in Table 2. Student's t-test and ANOVA were used for analysis of differences between average values. Weighted averages were used for sex difference comparisons. The right frontal SAS was 9.1% wider than the left and the difference was statistically significant (p<0.001). Correlation and regression analysis revealed strong interdependences between the right and left frontal SAS width: r = 0.81; p<0.0001. During the BOPT, the right frontal SAS was 4.8% wider than the left, although the difference remained statistically non-significant. Correlation and regression analysis revealed strong interdependences between the right and left frontal SAS width during the BOPT: r = 0.80; p<0.0001. Males had a significantly increased SAS width in the sitting position and during the BOPT (Figure 2).

**Table 2:** Mean values (± standard deviation) of sas-TQ (NIR-T/BSS) and cc-TQ (NIR-T/BSS) in the sitting position and during BOPT.

	Number	sas-TQ	cc-TQ
		(NIR-T/BSS)	(NIR-T/BSS)
Right frontal	38	10906	65.77
(sitting position)		±3293	±18.09
Left frontal	38	9997*	69.09^NS^
(sitting position)		±3025	±13.82
Right frontal	38	8648$	40.59$
(BOPT)		±2986	±15.13
Left frontal	38	8248* [Table-fn nt103]	43.70[Table-fn nt103]
(BOPT)		±2695	±14.26

* p<0.01 versus right sas-TQ in sitting position and during BOPT, respectively;

^$^p<0.001 versus sitting position;

NS not statistically-significant difference versus right cc-TQ.

### NIRT-B/SS (cc-TQ)

**Figure 3:**
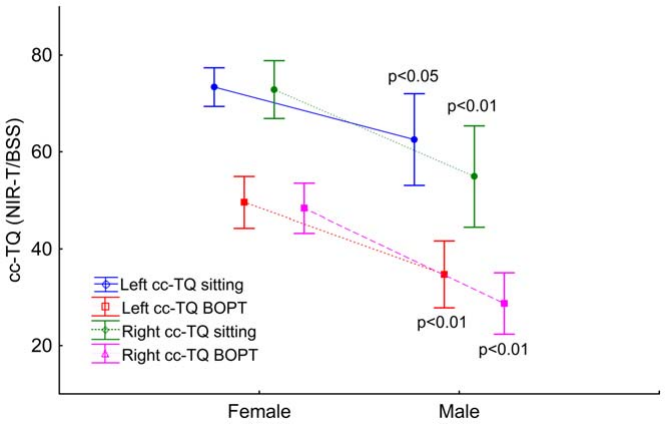
Dots and whisker plots show the mean left (blue) and right (green) pial artery pulsation (cc-TQ) in the sitting position, and left (red) and right (purple) pial artery pulsation (cc-TQ) during the BOPT measured with NIR-T/BSS in females (n = 23) and males (n = 15). The dots represent the means and the whiskers the 95% confidence intervals. Statistical significance (p values or NS for not statistically significant) are for sex asymmetries (females vs. males).

The numerical values of the left and right cc-TQ are presented in Table 2. Student's t-test and ANOVA were used for analysis of differences between average values. Weighted averages were used for sex difference comparisons. The left cc-TQ was 5.0% higher than the right, although the difference remained statistically non-significant. Correlation and regression analysis did not reveal any interdependence between the right and left cc-TQ. During the BOPT, the left cc-TQ was 7.7% higher than the right, although the difference was not statistically significant. Correlation and regression analysis revealed interdependence between the right and left cc-TQ during the BOPT: r = 0.53; p = 0.01. Females had a significantly increased cc-TQ in the sitting position and during the BOPT (Figure 3).

### MRI versus NIRT-B/SS (sas-TQ)

**Figure 4:**
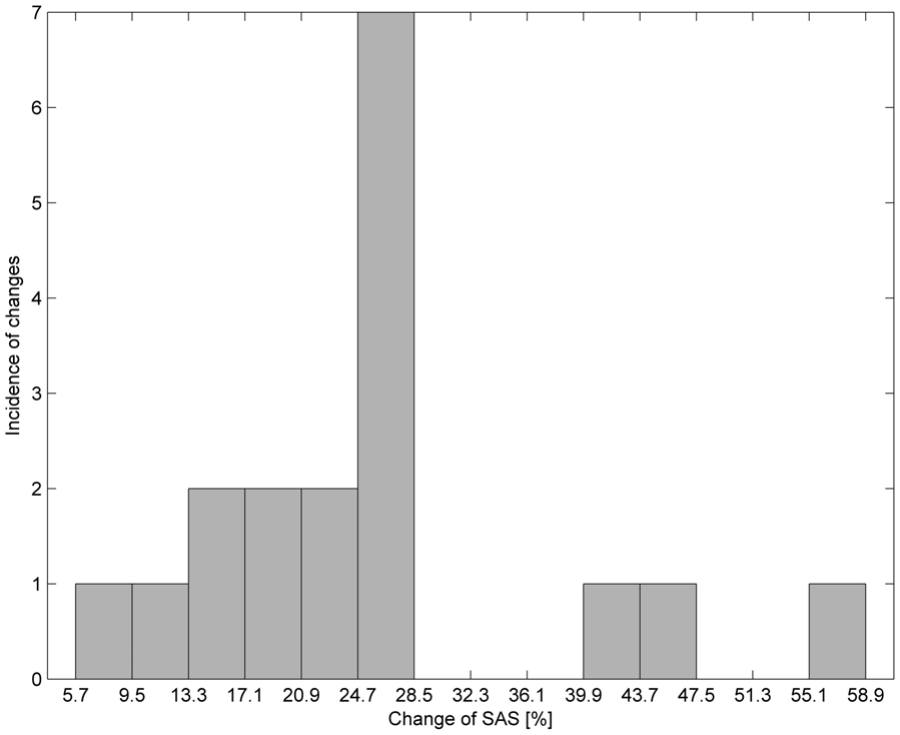
Histogram of percentage changes in results by switching from a lying-back position to an abdominal-lying position using the MRI method.

**Figure 5:**
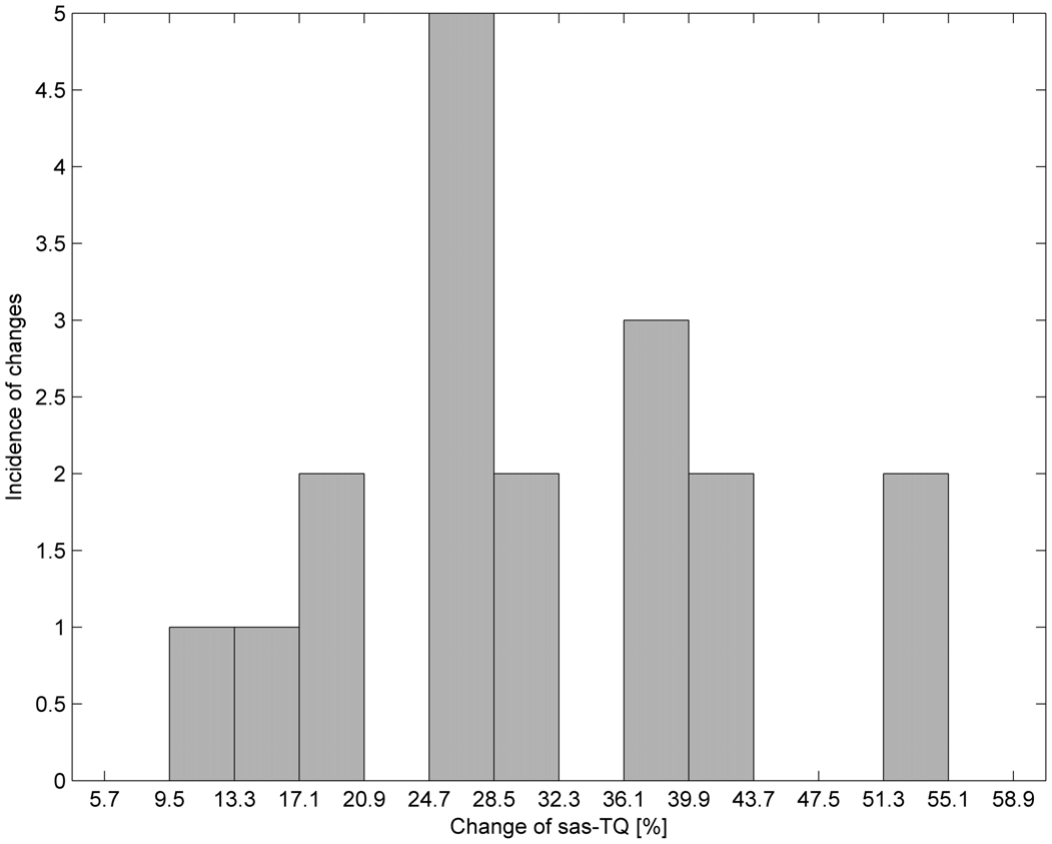
Histogram of percentage changes in results by switching from a lying-back position to an abdominal-lying position using the NIR-T/BSS method.

**Figure 6:**
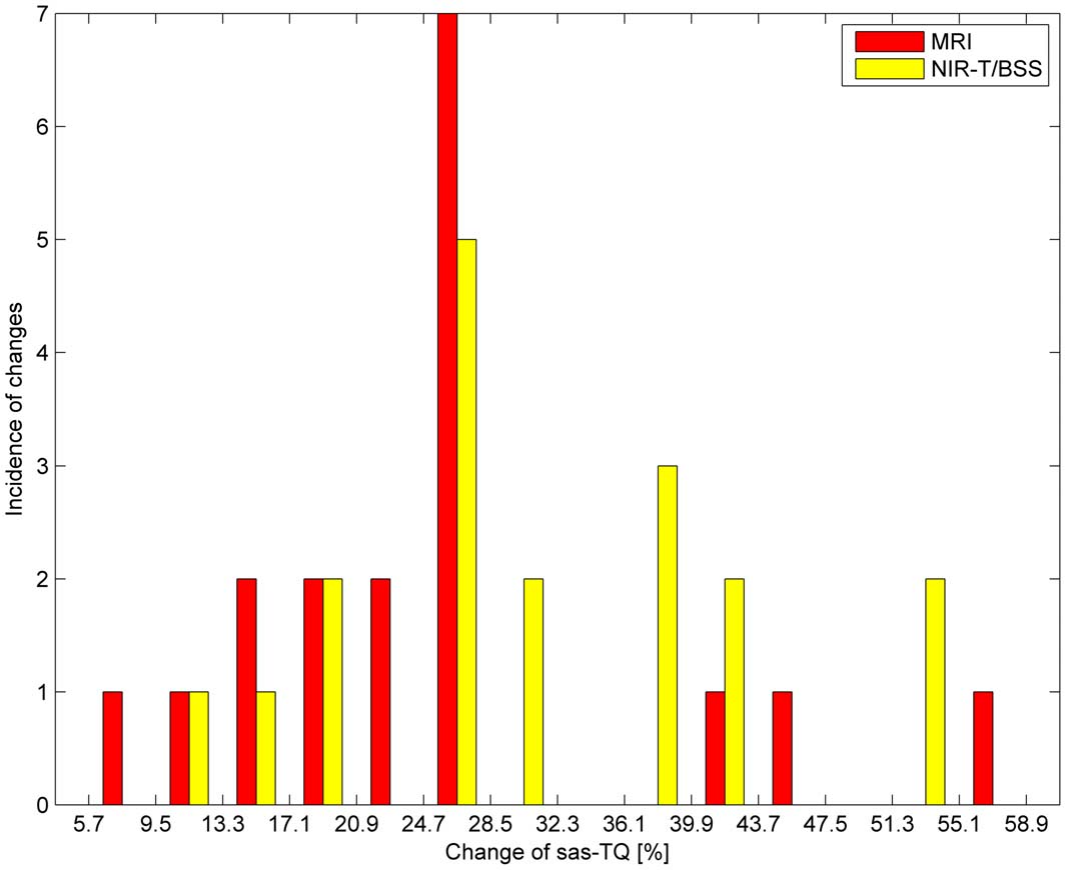
Histogram of percentage changes in results by switching from a lying-back position to an abdominal-lying position using both methods.

**Figure 7:**
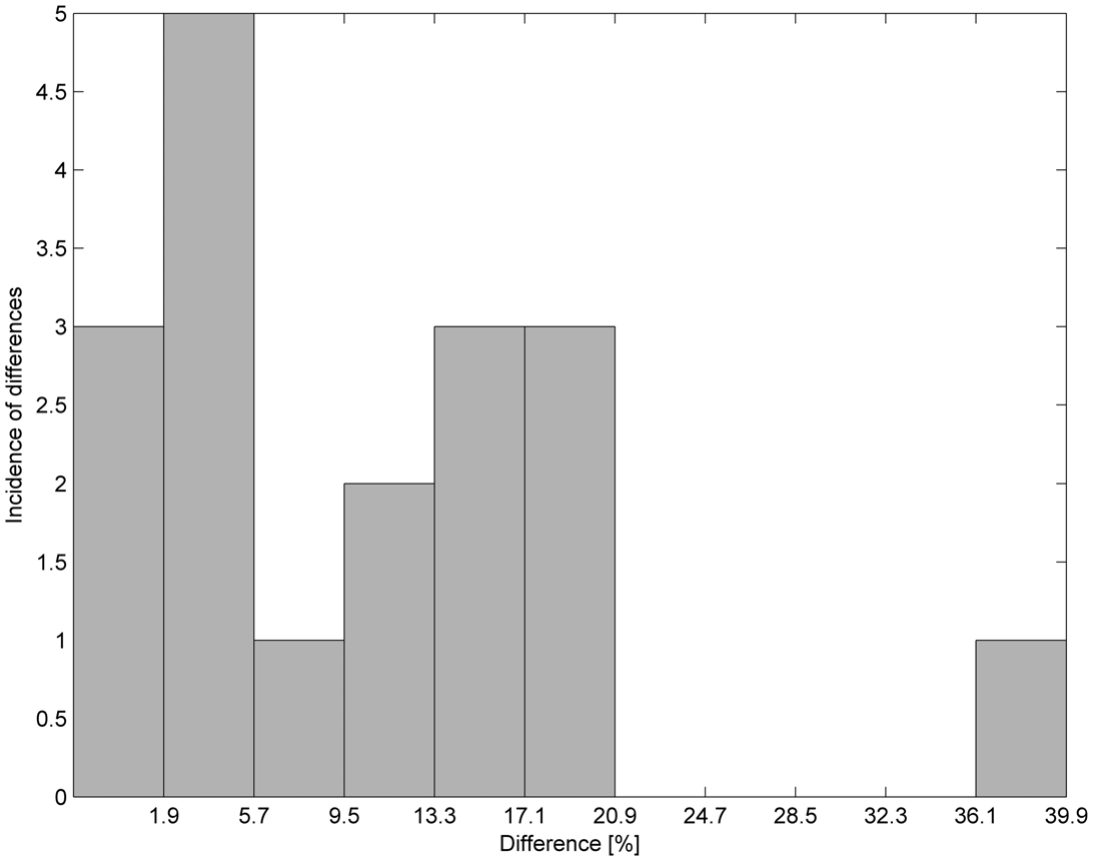
Histogram of differences between the percentage changes for MRI and NIR-T/BSS methods.

The measurement data included: width of the SAS in a lying-back position measured using the MRI method; width of the SAS in an abdominal-lying position measured using the MRI method; values of sas-TQ in a lying-back position measured using the NIR-T/BSS method; and values of sas-TQ in an abdominal-lying position measured using the NIR-T/BSS method. These data are presented in Table 3 and Table 4 and on the histograms in Figures 4, 5, 6, and 7.

**Table 3:** Measurement results using MRI method.

SAS measured in lying-back position [mm]	SAS measured in abdominal-lying position [mm]	Absolute change in SAS by switching position from back- to abdominal-lying [mm]	Absolute change in SAS by switching position from back- to abdominal-lying [%]
1.5800	1.1600	0.4200	26.5823
2.4200	1.9400	0.4800	19.8347
3.4200	2.8500	0.5700	16.6667
1.7800	1.5100	0.2700	15.1685
2.3500	1.7100	0.6400	27.2340
2.5300	1.3400	1.1900	47.0356
2.1400	1.5600	0.5800	27.1028
2.0400	1.8500	0.1900	9.3137
1.1800	0.9200	0.2600	22.0339
0.8500	0.6500	0.2000	23.5294
1.9000	1.1400	0.7600	40.0000
2.1600	0.9400	1.2200	56.4815
5.0300	4.0200	1.0100	20.0795
2.8300	2.0400	0.7900	27.9152
4.3700	3.2100	1.1600	26.5446
4.0900	3.5900	0.5000	12.2249
2.1100	1.5700	0.5400	25.5924
1.1800	0.8600	0.3200	27.1186

**Table 4:** Measurement results using NIR-T/BSS method.

sas- TQ measured in lying-back position	sas-TQ measured in abdominal-lying position	Absolute change in sas- TQ by switching position from back- to abdominal-lying	Absolute change in sas-TQ by switching position from back- to abdominal-lying [%]
14505	12585	1920	13.2368
13470	11205	2265	16.8151
10770	8085	2685	24.9304
5535	2685	2850	51.4905
9385	6760	2625	27.9702
11400	7824	3576	31.3684
4985	3120	1865	37.4122
4500	3300	1200	26.6667
6435	3780	2655	41.2587
5160	3135	2025	39.2442
5895	3420	2475	41.9847
8520	4129	4391	51.5376
8795	5480	3315	37.6919
10885	7480	3405	31.2816
14768	10836	3932	26.6251
15042	12358	2684	17.8434
12175	9101	3074	25.2485
14576	12057	2519	17.2818

The results of MRI are presented in the unit of millimetres, while the results of NIR-T/BSS are without any unit. Therefore, these two methods cannot be directly compared. However, it is possible to perform a statistical analysis of per cent changes in the results of each method when switching positions from a back- to abdominal-lying position.

In Figure 4 and 5, it can be seen that for both methods the most common changes are from 24.7–28.5%. Moreover, similarity in the occurrence of the changes for both methods can be observed by comparing the histograms in Figure 6. Table 5 contains the differences in percentage values of changes in the parameters measured using both methods. Figure 7 shows that all differences except one are within a limited range of less than 20.9%.

**Table 5:** Differences between the results for MRI method and NIR-T/BSS method.

Absolute change in SAS by switching position from back- to abdominal-lying [%]	Absolute change in sas- TQ by switching position from back- to abdominal-lying [%]	Absolute difference between changes for MRI and NIR-T/BSS [%]
26.5823	13.2368	13.3455
19.8347	16.8151	3.0196
16.6667	24.9304	8.2637
15.1685	51.4905	36.3220
27.2340	27.9702	0.7361
47.0356	31.3684	15.6672
27.1028	37.4122	10.3094
9.3137	26.6667	17.3529
22.0339	41.2587	19.2248
23.5294	39.2442	15.7148
40.0000	41.9847	1.9847
56.4815	51.5376	4.9439
20.0795	37.6919	17.6123
27.9152	31.2816	3.3664
26.5446	26.6251	0.0805
12.2249	17.8434	5.6184
25.5924	25.2485	0.3440
27.1186	17.2818	9.8368

**Figure 8:**
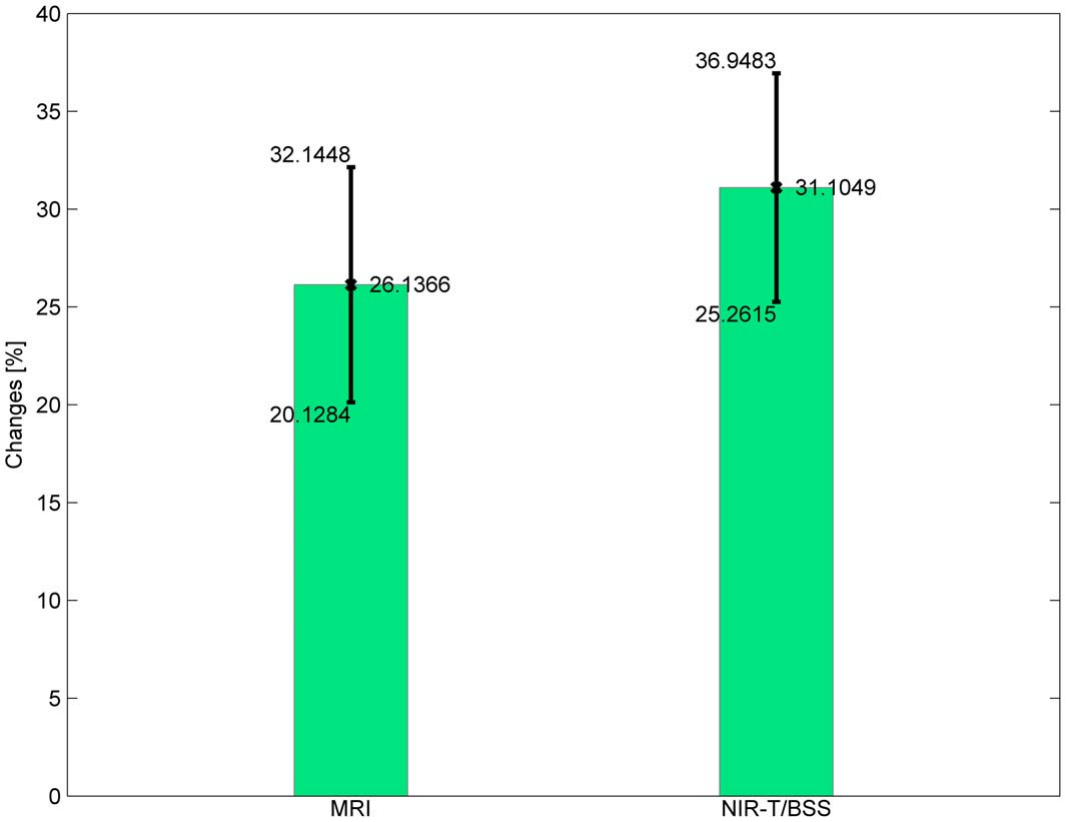
The mean value of percentage changes in results by switching from a lying-back to an abdominal-lying position using both methods.

The next stage of the analysis was to compare the mean changes of the changes measured by both methods. The mean value *μ* was calculated using this formula:
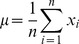
(1)where *n* is the sample size, 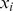 is the *i*-th result and 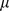 is the mean value of sample. In order to calculate the confidence interval of the mean value, the following model has been chosen: the characteristic of a population has any distribution of unknown parameters: i.e. the mean and finite variance. Sample size is 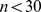 and confidence level is 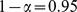. The confidence interval at level 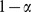 is determined by the following formulas:
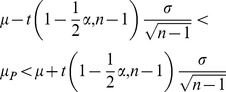
(2)

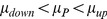
(3)

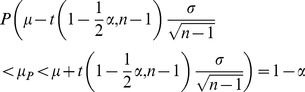
(4)where *μ* is the mean value from the sample, *σ* is the standard deviation of the sample, *n* is the sample size, 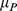 is the mean value of the population, 
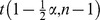
 is the quantile of Student's t-distribution with 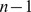 degrees of freedom. For a given confidence level, 
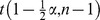
 is:
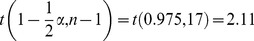
(5)For this model, the width of the confidence interval is:
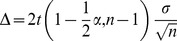
(6)
Tables 6, 7 and 8 and Figure 8 show that the NIRT method gives a higher mean value of changes in the measured values when switching the position of a tested patient. However, both calculated mean values are comparable and, more importantly, the width of confidence intervals are almost equal. Unfortunately, confidence intervals are relatively wide for both methods, but this is due to the small sample size.

**Table 6:** The mean value of changes in SAS by switching position from back- to abdominal-lying using the MRI method (calculated from data in Table 3).

	*μ*	*μ_down_*	*μ_up_*	Δ
[mm]	0.6167	0.4427	0.7907	0.3480
[%]	26.1366	20.1284	32.1448	12.0164

**Table 7:** The mean value of changes in sas-TQ by switching position from back- to abdominal-lying using the NIR-T/BSS method (calculated from data in Table 4).

	*μ*	*μ_down_*	*μ_up_*	Δ
[-]	2747.8333	2346.9292	3148.7375	801.8082
[%]	31.1049	25.2615	36.9483	11.6868

**Table 8:** The mean value of differences between the percentage changes in results for MRI and NIR-T/BSS methods (calculated from data in Table 5).

	*μ*	*μ_down_*	*μ_up_*	Δ
[%]	10.2079	5.4858	14.9301	9.4443

The next stage of the analysis was to compare the variance of the changes measured by both methods. The variance 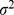 was calculated using this formula:
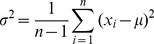
(7)where *n* is the sample size, 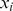 is the *i*-th result, *μ* is the mean value of sample and 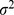 is the variance of sample (Tables 9, 10 and 11).

**Table 9:** The variance of changes in SAS by switching position from back- to abdominal-lying using the MRI method (calculated from data in Table 3).

	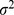
For quantitative changes	0.1156
For percentage changes	137.8384

**Table 10:** The variance of changes in sas-TQ by switching position from back- to abdominal-lying using the NIR-T/BSS method (calculated from data in Table 4).

	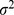
For quantitative changes	613712.6176
For percentage changes	130.3814

**Table 11:** The variance of differences between the percentage changes in results for MRI and NIR-T/BSS methods (calculated from data in Table 5).

	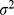
For percentage changes	85.1457

**Figure 9:**
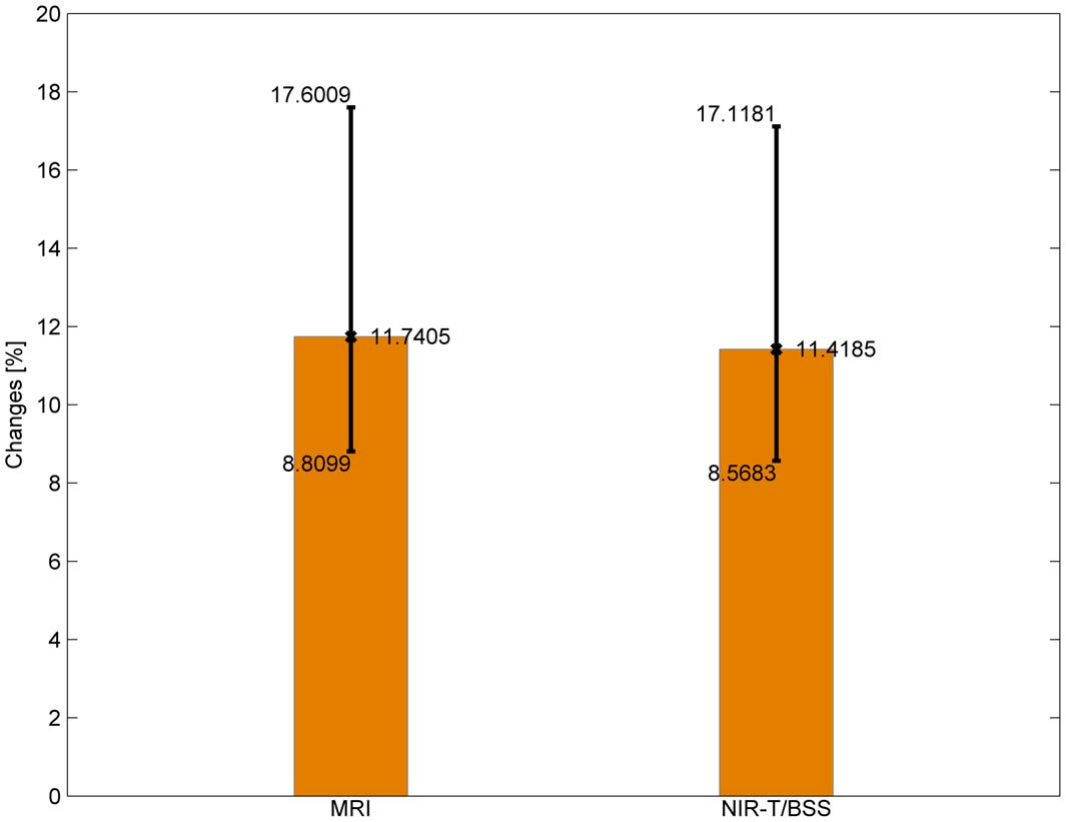
The standard deviation of percentage changes in results when switching from a lying-back position to an abdominal-lying position using both methods.

The next stage of the analysis was to compare the standard deviations of the changes measured by both methods. The standard deviation σ was calculated using this formula:
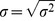
(8)where 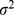 is the variance of sample and σ is the standard deviation of sample. In order to calculate the confidence interval of the standard deviation, the following model has been chosen: the characteristic of a population has normal distribution of unknown parameters: i.e. the mean and the variance. Sample size is 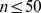 and confidence level is 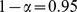. The confidence interval at level 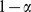 is determined by the following formulas:
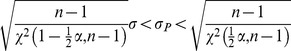
(9)

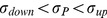
(10)

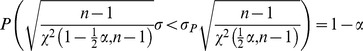
(11)where *σ* is the standard deviation of the sample, *n* is the sample size, 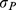 is the standard deviation of the population, 
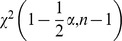
 and 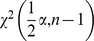 are quantile of 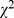 distribution with 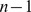 degrees of freedom. For a given confidence level, they are:
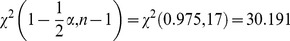
(12)

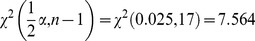
(13)For this model, the width of the confidence interval is:
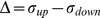
(14)In Tables 12, 13, 14 and Figure 9 it can be observed that the standard deviations of changes in the measured values, as well as confidence intervals are almost the same for both examined methods. Confidence intervals for the standard deviation are asymmetric, which results from the model used, which was dictated by the sample size.

**Table 12:** The standard deviation of changes in SAS by switching position from back- to abdominal-lying using the MRI method (calculated from data in Table 3).

	*σ*	*σ_down_*	*σ_up_*	Δ
[mm]	0.3400	0.2551	0.5097	0.2546
[%]	11.7405	8.8099	17.6009	8.7910

**Table 13:** The standard deviation of changes in sas-TQ by switching position from back- to abdominal-lying using the NIR-T/BSS method (calculated from data in Table 4).

	*σ*	*σ_down_*	*σ_up_*	Δ
[mm]	783.3981	587.8523	1174.4411	586.5887
[%]	11.4185	8.5683	17.1181	8.5499

**Table 14:** The standard deviation of differences between the percentage changes in results for MRI and NIR-T/BSS methods (calculated from data in Table 5).

	*σ*	*σ_down_*	*σ_up_*	Δ
[%]	9.2274	6.9242	13.8334	6.9093

The next stage of the analysis was to compare the Mean-Square Error, Root-Mean-Square Error and Normalized Root-Mean-Square Error of the changes measured by both methods. Root-Mean-Square Error is usually the most important measure, but the tested values are not expressed in the same unit, so the most important measure is the Normalized Root-Mean-Square Error. Mean-Square Error *MSE* was calculated using this formula:
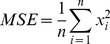
(15)where *n* is the sample size, 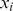 is the *i*-th result and *MSE* is the Mean-Square Error.

Root-Mean-Square Error *RMSE* was calculated using this formula:
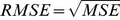
(16)where *MSE* is the Mean-Square Error and *RMSE* is the Root-Mean-Square Error.

Normalized Mean-Square Error *NRMSE* was calculated using this formula:
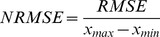
(17)where 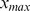 is the maximum result of the sample, 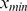 is the minimum result of sample and *NRMSE* is Normalised Mean-Square Error (Tables 15, 16 and 17).

**Table 15:** MSE, RMSE and NRMSE of changes in SAS by switching position from back- to abdominal-lying using the MRI method (calculated from data in Table 3).

	MSE	RMSE	NRMSE
For quantitative changes	0.4895	0.6996	0.6792
For percentage changes	813.3016	28.5184	0.6046

**Table 16:** MSE, RMSE and NRMSE of changes in sas-TQ by switching position from back- to abdominal-lying using the NIR-T/BSS method (calculated from data in Table 4).

	MSE	RMSE	NRMSE
For quantitative changes	8130205.5000	2851.3515	0.8936
For percentage changes	1090.6514	33.0250	0.8623

**Table 17:** MSE, RMSE and NRMSE of differences between the percentage changes in results for MRI and NIR-T/BSS methods (calculated from data in Table 5).

	MSE	RMSE	NRMSE
For percentage changes	184.6176	13.5874	0.3749

The correlation coefficient *r* between changes in SAS measured by the MRI method and changes in sas-TQ measured by the NIR-T/BSS method while switching from a laying-back position to an abdominal-lying position was calculated using this formula:
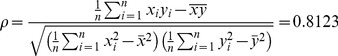
(18)where *n* is the sample size, 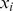 is the *i*-th result for the NIR-T/BSS method (Table 4), 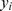 is the *i*-th result for the MRI method (Table 3), 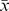 is the mean value of the sample for the NIR-T/BSS method (Tab.7), 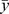 is the mean value of the sample for the MRI method (Table 6). The correlation coefficient is high (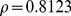), which indicates a strong correlation between the results obtained for both methods (Table 18).

**Table 18:** The correlation coefficient between changes in SAS measured by the MRI method and changes in sas-TQ measured by the NIR-T/BSS method while switching from back- to abdominal-lying position.

*ρ*	*ρ_down_*	*ρ_up_*	Δ
0.8123	0.5580	0.9275	0.3695

**Figure 10:**
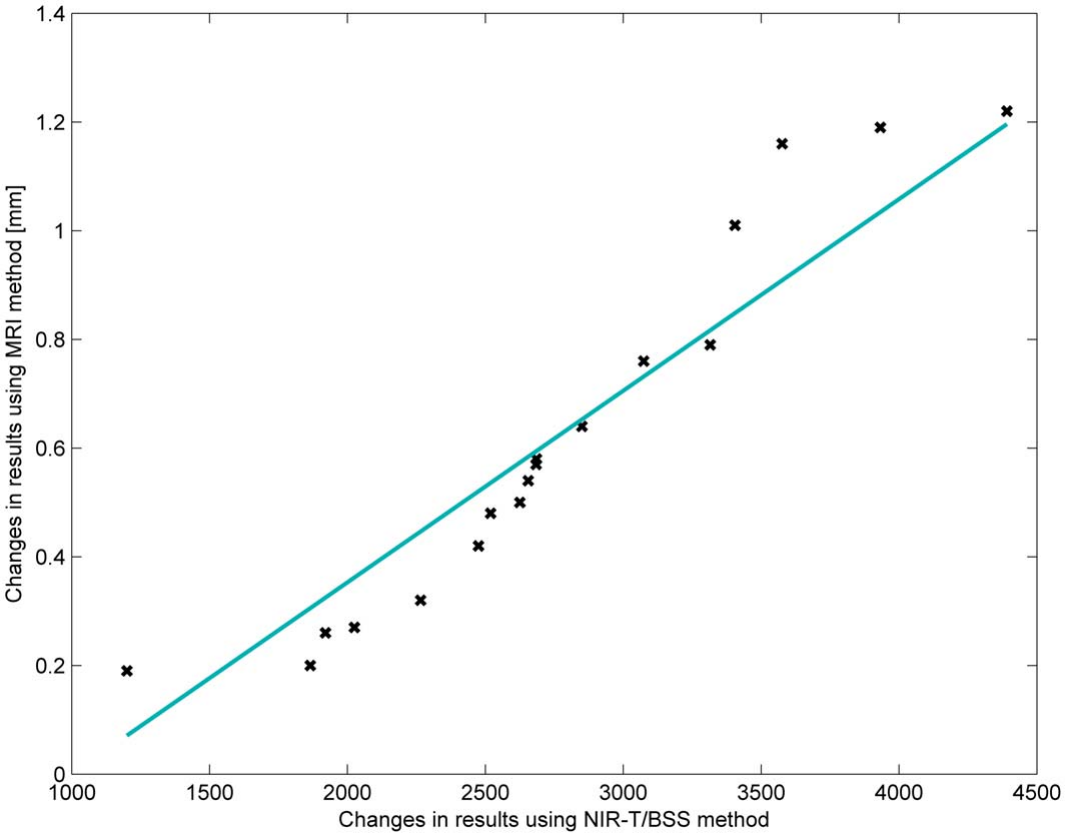
Correlation diagram of changes in results when switching from a lying-back position to an abdominal-lying position using both methods.

In order to calculate the confidence interval of the correlation coefficient, the following model has been chosen: the characteristic of a population has normal distribution 
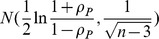
. Sample size is 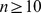 and confidence level is 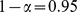. The confidence interval at level 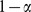 is determined by the following formulas:
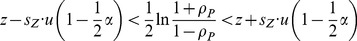
(19)

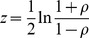
(20)

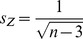
(21)

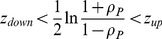
(22)

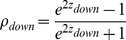
(23)

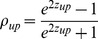
(24)

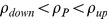
(25)

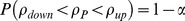
(26)where *ρ* is the correlation coefficient from sample, *n* is the sample size, 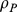 is the correlation coefficient of the population, 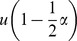 is the quantile of 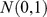. For a given confidence level, 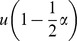 is:
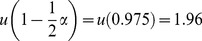
(27)For this model, the width of the confidence interval is:
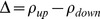
(28)Regression function was calculated using the following formulas:
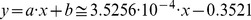
(29)

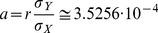
(30)

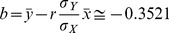
(31)where 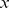 is a result for the NIR-T/BSS method, 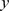 is a result for the MRI method, *a* is the coefficient of linear regression, *b* is the shift factor, 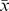 is the mean value of the sample for the NIR-T/BSS method, 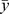 is the mean value of the sample for the MRI method, 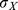 is the standard deviation of the sample for the NIR-T/BSS method, 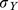 is the standard deviation of the sample for the MRI method. The regression function is shown in Figure 10 as a blue line.

**Figure 11:**
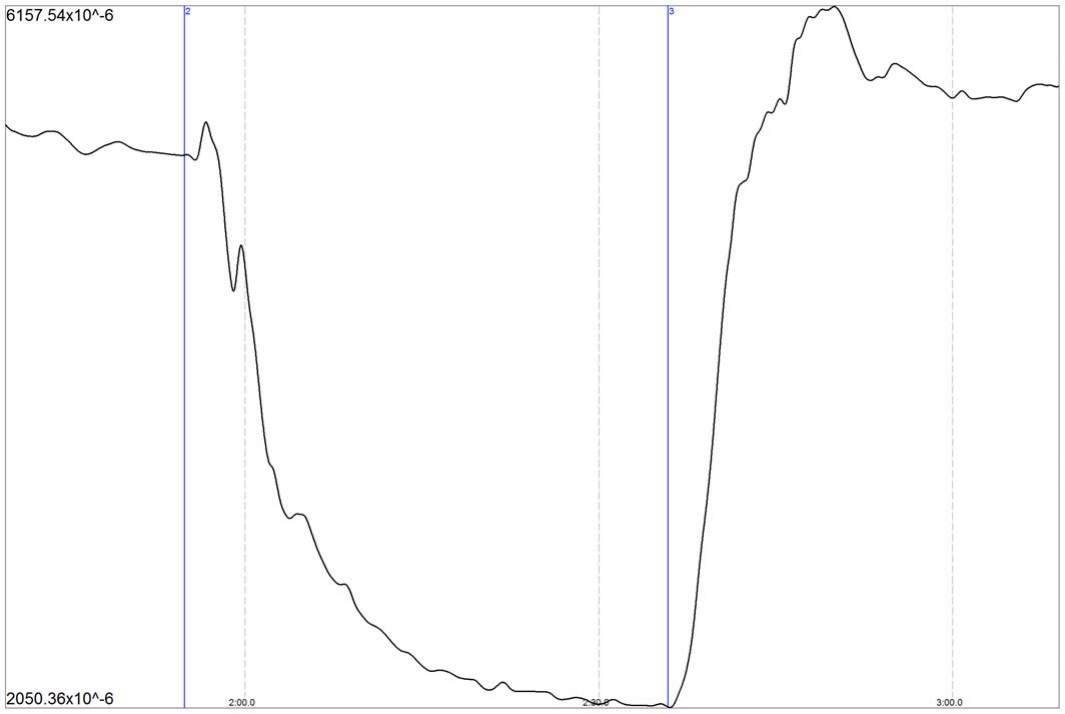
An NIR-T/BSS tracing demonstrating the sas-TQ change between the back- and abdominal-lying positions. The first marker indicates the change from the back- to the abdominal-lying position, while the second marker indicates the change from the abdominal- to the back-lying position.

Figure 11 shows a representative NIR-T/BSS tracing with sas-TQ changes between the back- and abdominal-lying positions.

## Discussion

There are several new findings from this study: (1) the NIRT-B/SS recordings are comparable and equivalent to MRI measurements and consistent with historical MRI data from the literature; (2) the frontal SAS width (sas-TQ) is larger in males and in the right hemisphere; (3) pial artery pulsation is higher in females and also likely to be in the left hemisphere. Furthermore, this study establishes a reference for further research conducted in patients with intracranial pathologies such as brain tumours and carotid artery stenosis, to name a few.

Cerebral left-right asymmetry and sexual dimorphism in the human brain have been reported in several MRI studies [21]–[23]. However, to the best of our knowledge, this is the first such study focusing on the SAS. Although the SAS is sometimes measured in children for diagnostic purposes [1], [2], [24], there has been no reason to investigate the SAS in adults to date. The emergence of NIRT-B/SS technology enables the SAS width to be measured easily at a low cost [10], [11], [13]. We assume that if the right hemisphere is larger, the SAS width and subsequently sas-TQ should also be bigger. The same rule should apply to sexual dimorphism. Therefore, the NIRT-B/SS sas-TQ results were fully consistent with the current MRI examination and historical data [6], [21]–[23]. It should nonetheless be noted that the differences between hemispheres seen during MRI examination in the present study, although identical with that previously reported, were not statistically significant. The most probable explanation for such results is the small sample size. The above-cited studies included much larger subject populations. Although the limited number of subjects investigated with MRI can be seen as a limitation, MRI SAS width assessment was performed in the first instance to demonstrate that the SAS can be easily measured in adults. To the best of our knowledge, only one study was performed earlier to assess the SAS width in adults [6]. However, it was not our aim to repeat the earlier morphological measurements [21]–[23]. The NIR-T/BSS group was larger and yielded statistically significant differences between the hemispheres, consistent with the literature data.

It has been demonstrated earlier that cc-TQ reflects changes in pial artery compliance. cc-TQ increases have been observed during acetazolamide and hypercapnic tests, acute hypoxia, papaverine and glucagon administration and electroconvulsive therapy, while cc-TQ decreases have been recorded during handgrip tests [14] and the stabilisation period after the abovementioned procedures [10], [12], [17], [18]. The finding that cc-TQ is higher in females is in line with the evidence reported by other authors. Early studies indicated that women have a higher resting mean cerebral blood flow compared with men [25], [26]. Later studies have shown, sex-related differences in cerebral blood flow have been attributed to changes in arterial tone. Oestrogen decreases whereas testosterone increases the vascular tone of cerebral arteries. Therefore, one of the major influences of sex steroid hormones on the cerebral vasculature is the ability to alter vascular reactivity and thereby modulate blood flow [27]. The high sensitivity of cc-TQ as an index of pial arteries compliance potentially provides a new tool to investigate vascular adaptations during pregnancy and changes in cerebral vessels reactivity implicated in eclampsia, a leading cause of maternal death [28]. In the current study, we did not assess the influence of hemisphere dominance on pial artery pulsation. cc-TQ was slightly higher in the left hemisphere, in accordance with the evidence that cerebral blood flow is higher in the left hemisphere [29]. Functional hemispheric asymmetry can be investigated by simultaneous cerebral blood flow velocity monitoring with transcranial Doppler [30]. A well-designed study is needed to clarify if NIR-T/BSS cc-TQ modality can be used for such purposes.

The brain, enclosed in the in-distensible skull, is subject to gravitation and changes its position along with changes in the position of the head [31]. At a forward bending position (during BOPT) or in the abdominal-lying position, the brain floats forward toward the inner surface of the frontal bones and the SAS in the frontal region assumes its minimum value. The BOPT, described earlier in detail, evokes very typical changes in sas-TQ and cc-TQ due to physical brain movements [10], [14]. Nevertheless, here we compared for the first time simultaneous sas-TQ and cc-TQ changes in both hemispheres. The correlation between cc-TQ in both hemispheres observed during BOPT probably reflects the influence of similarly increased intracranial pressure on pial arteries on both sides. This is a weak correlation and most likely without real clinical significance. However, it may suggest that cc-TQ is modulated by even very slight increases in intracranial pressure, which is in agreement with data from earlier animal studies with NIR-T/BSS [10], [13]. Interestingly, sas-TQ in the right and left hemispheres correlated to each other in the sitting position and during BOPT. As the same correlation was observed between the SAS widths in MRI, we may consider this finding as an additional indirect proof that with regards to the SAS width, MRI and NIR-T/BSS recordings are equivalent. Furthermore, the direction and range of the sas-TQ decrease observed during BOPT in this study was very similar to those demonstrated earlier [10], [14], thus confirming the high repeatability of the NIR-T/BSS results.

It is very encouraging to see that the parameters measured in this NIR-T/BSS study matched well with the MRI data in the literature, even though different groups of subjects were used for these two different modalities. Nevertheless, comparison to historical or literature data is usually considered to be relatively weak. Therefore, we performed head-to-head comparison between MRI and NIR-T/BSS measurements. The change in SAS width between the back- and abdomen-lying positions was analysed with by MRI and NIR-T/BSS. The number of volunteers was limited as lying in the abdominal position in MRI is extremely unpleasant. Nevertheless, we examined 9 subjects, which meant that we could compare data from 18 hemispheres. The direct comparison between MRI and NIRT-B/SS using the same group of subjects (hence eliminating intra-subject variation) and an identical procedure (changing from the back- to the abdominal-lying position) yielded very good quantitative agreement between both methods (Figure 10). The relationship between changes in the results for the MRI method and changes in the results of the NIR-T/BSS method, when switching from the lying-back position to the abdominal-lying position, can be approximated by a linear relationship with a certain finite accuracy. The conclusion of this analysis is that the results from both methods are highly correlated with each other and it is possible to develop an equation that, with predefined precision, will allow for a two-way exchange of values obtained by the two examined methods. This direct comparison further supports and validates NIRT-B/SS technology in SAS width measurements. We did not observe any interdependence between MRI and NIR-T/BSS in the back-lying or abdominal-lying position (data not shown). This is however not surprising, as NIR-T/BSS demonstrates high repeatability when the relative changes are analysed [9], [10], [12]–[14], [18], [19], [32]. So far, the measurements using IR light (NIRS and NIR-T/BSS) does not allow for direct between-subject comparisons due to differences in skull bone parameters [10], [33]. NIR-T/BSS can be potentially used for screening SAS width and/or pial artery asymmetry, allowing for early detection of intracranial abnormalities in ambulatory (general practice) conditions. The sas-TQ and MRI results reported in this study, as well as historical differences between the hemispheres, were below 10%. It seems likely that the SAS width asymmetry in patients with brain tumours should far exceed 10% and thus be detected in sas-TQ measurements as a non-physiological parameter requiring further attention. The SAS width has not been investigated in patients with brain tumours yet. Taking into account the presented results, such a study is warranted. Moreover, the BOPT test, developed 10 years ago [10], can be potentially used to quickly check the brain expansion reserve, for instance in patients with brain oedema and/or increased intracranial pressure. In physiological conditions, sas-TQ decreases during the BOPT by 20% to 30%. In patients with increased intracranial pressure, for example due to brain oedema, such a decrease is not seen (unpublished data from our lab). Differences in skull bone parameters are negligible as long as the same subject is analysed or diagnosed. The current study and head-to-head comparison between the MRI and NIR-T/BSS establishes the reference for future planned studies in patients with brain tumours and increased intracranial pressure. Furthermore, NIR-T/BSS, due to low cost and non-invasiveness, potentially offers the possibility of long-term treatment follow-up.

We demonstrated that NIR-T/BSS sas-TQ and MRI are comparable and equivalent modalities for SAS width change measurements. Moreover, sas-TQ and cc-TQ values were consistent with the literature data related to sexual dimorphism and morphological asymmetries between the hemispheres. NIR-T/BSS constitutes a potentially cheap and easy-to-use method for early screening in patients with brain tumours, increased intracranial pressures and other abnormalities. Further studies in patients with intracranial pathologies are warranted.
